# Helicase of Type 2 Porcine Reproductive and Respiratory Syndrome Virus Strain HV Reveals a Unique Structure

**DOI:** 10.3390/v12020215

**Published:** 2020-02-14

**Authors:** Chenjun Tang, Zengqin Deng, Xiaorong Li, Meiting Yang, Zizi Tian, Zhenhang Chen, Guoguo Wang, Wei Wu, Wen-hai Feng, Gongyi Zhang, Zhongzhou Chen

**Affiliations:** 1 State Key Laboratory of Agrobiotechnology and Beijing Advanced Innovation Center for Food Nutrition and Human Health, College of Biological Sciences, China Agricultural University, Beijing 100193, China; tcj_0814@cau.edu.cn (C.T.); dzq@cau.edu.cn (Z.D.); xiaorongli@cau.edu.cn (X.L.); meitingyang@cau.edu.cn (M.Y.); sz20153020048@cau.edu.cn (Z.T.); sz11060758@cau.edu.cn (Z.C.); sz20193020193@cau.edu.cn (G.W.); wuweiyou@cau.edu.cn (W.W.); whfeng@cau.edu.cn (W.-h.F.); 2 Department of Biomedical Research, National Jewish Health, Denver, CO 80206, USA; zhangg@njhealth.org

**Keywords:** porcine reproductive and respiratory syndrome virus, crystal structures, helicase, novel domain, zinc-binding domain, SAXS

## Abstract

Porcine reproductive and respiratory syndrome virus (PRRSV) is prevalent throughout the world and has caused great economic losses to the swine industry. Nonstructural protein 10 (nsp10) is a superfamily 1 helicase participating in multiple processes of virus replication and one of the three most conserved proteins in nidoviruses. Here we report three high resolution crystal structures of highly pathogenic PRRSV nsp10. PRRSV nsp10 has multiple domains, including an N-terminal zinc-binding domain (ZBD), a β-barrel domain, a helicase core with two RecA-like domains, and a C-terminal domain (CTD). The CTD adopts a novel fold and is required for the overall structure and enzymatic activities. Although each domain except the CTD aligns well with its homologs, PRRSV nsp10 adopts an unexpected extended overall structure in crystals and solution. Moreover, structural and functional analyses of PRRSV nsp10 versus its closest homolog, equine arteritis virus nsp10, suggest that DNA binding might induce a profound conformational change of PRRSV nsp10 to exert functions, thus shedding light on the mechanisms of activity regulation of this helicase.

## 1. Introduction

Porcine reproductive and respiratory syndrome (PRRS) is characterized by reproductive failure in sows and respiratory diseases in piglets [1,2]. This disease is one of the most infectious diseases in the swine industry worldwide and has brought great economic losses since it was first reported in the late 1980s [3,4]. The etiological agent, porcine reproductive and respiratory syndrome virus (PRRSV), is a single positive-stranded RNA virus, which is classified into two genotypes: the type 1 PRRSV (PRRSV-1) and type 2 PRRSV (PRRSV-2) with approximately 40% difference at the nucleotide level [5,6]. Recently, PRRSV-1 and PRRSV-2 have been taxonomically classified into the species *Betaarterivirus suid 1* and *2* belonging to the genus *Betaarterivirus* of the family *Arteriviridae* in the order *Nidovirales* [7]. The PRRSV genome is approximately 15.4 kb in length and includes at least 12 open reading frames (ORFs). The ORF1a and ORF1b encode polyproteins pp1a and pp1ab, which are subsequently processed into many functional nonstructural proteins (nsps) essential for virus replication, genomic transcription, viral pathogenesis, and virulence [8,9,10,11,12].

In 2006, a large-scale atypical PRRS outbreak caused by the highly pathogenic PRRSV (HP-PRRSV, belonging to PRRSV-2) emerged in China [13,14]. Yan Li and her colleagues demonstrated that nsp9 and nsp10 together contributed to the replication efficiency and the high virulence of HP-PRRSV [15]. Nsp9 contains an RNA-dependent RNA-polymerase (RdRp) domain [16]. Nsp10 belongs to the superfamily 1B (SF1B) Upf1-like family of helicases, which could unwind both DNA and RNA duplexes [17,18,19]. In addition, this family of helicases contains an N-terminal predicted zinc-binding domain (ZBD) which is conserved in all nidovirus helicases, including 12 or 13 conserved Cys and His residues [20]. However, the C-terminal domain (CTD) is variable among nidoviruses. Besides, nsp10 can vary up to approximately 42% on the amino acid level between the two genotypes, and the strains within PRRSV-2 also vary considerably with amino acid differences as high as 8% [21,22]. We previously reported the first structure of the nidovirus helicase, equine arteritis virus (EAV; family *Arteriviridae*) nsp10, and demonstrated that the CTD perhaps exerts a regulatory function on the helicase core, facilitating coupling between NTPase and polynucleotide binding activities [23]. Another solved structure of nidovirus helicase is Middle East respiratory syndrome coronavirus (MERS-CoV; family *Coronaviridae*) nsp13 [24]. While the domain organization of nsp13 is similar to EAV nsp10, structural comparisons of the individual domains showed that nsp13 is closely related to Upf1. The N-terminal Cys-His-rich domain (CH domain) of nsp13 is more related to the CH of Upf1 than to the ZBD of EAV nsp10. The helicase core of EAV nsp10 is more compact than that of MERS-CoV nsp13 which has a similar size as Upf1. Meanwhile, MERS-CoV nsp13 does not contain a C-terminal regulatory domain homologous to the CTD of EAV nsp10. 

Reflecting its importance, PRRSV nsp10 is considered to be a major drug target. However, despite extensive efforts, the three-dimensional structure of PRRSV nsp10 remained unresolved, which brought about the difficulty to structure-based drug design. In this paper, we present the structure of HP-PRRSV nsp10 in full length and truncation, which has a different domain arrangement compared with other helicases. From our structures, combined with structural and biochemical analyses, a number of interesting features are revealed. Firstly, we present the first structural insight into the CTD of nidovirus helicases. Secondly, our structures demonstrate that PRRSV nsp10 adopts an unexpected extended overall structure in crystals and solution. Thirdly, structural and functional analyses suggest that DNA binding might induce a profound conformational change of PRRSV nsp10.

## 2. Materials and Methods

### 2.1. Cloning, Expression, and Purification of PRRSV nsp10

The DNAs encoding full-length PRRSV nsp10 were amplified from the cDNAs of the HP-PRRSV strain HV (GenBank Accession number JX317648.1) [25] and low pathogenic PRRSV (LP-PRRSV) strain CH-1R (GenBank Accession number EU807840.1) [26] and ligated into a modified pET-28a in which the thrombin recognition site was replaced by a tobacco etch virus (TEV) protease recognition site [27]. The DNA encoding the C-terminally truncated version comprising residues 1–366 (nsp10Δ) was amplified from the cDNA of the PRRSV strain HV (GenBank Accession number JX317648.1) [25] and inserted into a modified pET-28a encoding a cleavable N-terminal small ubiquitin-like modifier (SUMO) tag. Point mutations were introduced on nsp10 expression constructs using site-directed PCR-based mutagenesis and subcloning. The method of protein expression was similar to the previous publication [23]. The cultures were centrifuged and cell pellets were collected and resuspended in lysis buffer (20 mM HEPES pH 7.0, 500 mM NaCl) and then disrupted by sonication. The lysate was centrifuged at 47,000× *g* for 30 min to remove cell debris. The soluble fraction was applied to a Ni^2+^ chelating resin. After sample loading, the resin was washed with washing buffer (20 mM HEPES pH 7.0, 500 mM NaCl and 40 mM imidazole). The protein was eluted with elution buffer (20 mM HEPES pH 7.0, 500 mM NaCl and 200 mM imidazole). After changing the buffer to remove the imidazole, the protein was digested with 10% (*w*/*w*) TEV protease at 4 °C overnight to remove N-terminal His-tag. Further purification was performed by size-exclusion chromatography (SEC) using Superdex 75 10/300 GL (GE Healthcare, Uppsala, Sweden). Peak fractions were collected and analyzed by SDS-PAGE. 

### 2.2. Reductive Methylation

The protein eluted from Ni^2+^ chelating resin was diluted into buffer (50 mM HEPES pH 7.6, 500 mM NaCl) and concentrated to 1 mg/mL. For each 1 mL of protein, 10 µL 1 M borane dimethylamine complex was added followed by 20 µL 1 M formaldehyde [28]. The addition of borane dimethylamine complex and formaldehyde was repeated after 2 h. After another 2 h, 5 µL 1 M borane dimethylamine complex was added and the reaction was incubated overnight. To terminate the reaction, Tris-HCl pH 8.0 was added to the solution to 100 mM. The methylated protein was subsequently purified by gel filtration using the same conditions as used for holo PRRSV nsp10. The peak fractions were analyzed by SDS-PAGE. 

### 2.3. DNA Preparations

All DNA oligonucleotides were synthesized from Invitrogen (Shanghai, China). DNA duplexes used for crystallization (top strand: 5′–TTTTTTTTTTCGAGCACCGCTGCGGCTGCACC–3′, bottom strand: 5′–GGTGCAGCCGCAGCGGTGCTCG–3′), electrophoretic mobility shift assay (EMSA) and ATPase assay (top strand: 5′–TTTTTTTTTTCGAGCACCGCTGCGGCTG*–3′, bottom strand: 5′–GGTGCAG CCGCAGCGGTGCTCG–3′, asterisk indicates the position of the FAM label), and unwinding assay (top strand: 5′–TTTTTTTTTTGCCTCGCTGCCGTCGCCACC–3′, bottom strand: 5′–*GGTGGCGAC GGCAGCGAGGC–3′, asterisk indicates the position of the FAM label) were dissolved in the buffer containing 10 mM Tris-HCl pH 8.0 and 100 mM NaCl. Annealing was performed by heating the mixture at 95 °C for 5 min and slowly cooling to room temperature in 2 h.

### 2.4. Crystallization and Data Collection

To obtain the crystals of HP-PRRSV nsp10, 7 mg/mL methylated nsp10 was mixed with a well solution containing 28% PEG 400, 0.1 M HEPES pH 7.5 and 0.2 M CaCl_2_. To obtain crystals of the nsp10-DNA complex, purified nsp10 and partially double-stranded DNA (dsDNA) described above or single-stranded DNA (ssDNA) used for Small-angle X-ray scattering (SAXS) measurement of the nsp10-DNA complex (5’–TTTTTTTTTTCGAGCACCGCTGCGGCTG–3’) were mixed in a 1:1.2 molar ratio and incubated at 4 °C overnight. The condition of crystals of full length nsp10 in the presence of DNA was the same as the methylated nsp10. Crystals of the truncated nsp10 were achieved in reservoir buffer containing 10% (*v*/*v*) PEG 5000 MME, 12% (*v*/*v*) 1-propanol, 0.1 M MES pH 6.5. For data collection, crystals were cryoprotected in mother liquor supplemented with 20% (*v*/*v*) glycerol and flash cooled to −173 °C. 

X-ray diffraction data of PRRSV nsp10 were collected on beamlines BL17U and BL18U at the Shanghai Synchrotron Radiation Facility (SSRF), and were processed with HKL2000 [29]. Data collection and processing statistics are shown in Table 1.

### 2.5. Structure Determination

The structure of methylated PRRSV nsp10 was solved by single wavelength anomalous dispersion (SAD) method, with three zinc ions per asymmetric unit. The initial phase was obtained by the program autoSHARP [30]. The figure of merit from the SAD phasing was 0.19. A crude partial model was traced automatically using the program Buccaneer [31]. The final resulting map was in good quality to clearly show the molecular boundary and α-helix bundles. The model was built manually in the program COOT [32], and refinement was carried out with REFMAC5 [33]. The structures of holo nsp10 and truncated nsp10 were solved by molecular replacement using methylated PRRSV nsp10 as the search model. The initial models were obtained by MOLREP and Balbes, respectively [34,35]. Data refined parameters are summarized in Table 1. Atomic coordinates and structure factors for the reported crystal structures have been deposited with the Protein Data Bank (PDB) under accession numbers 6JDR, 6JDU, and 6JDS. All figures in this article displaying molecular structure were made using PYMOL [36].

### 2.6. NTPase Assay

ATPase activity was measured using a direct colorimetric assay as previously described [37,38]. Briefly, in a 20 μL reaction volume, 0.02 μM nsp10, 0.2 μM partially dsDNA (top strand: 5′–TTTTTTTTTTCGAGCACCGCTGCGGCTG–3′, bottom strand: 5′–GGTGCAGCCGCAGCGGTGC TCG–3′), 40 mM HEPES pH 7.5, 2.5 mM MgCl_2_, 2 mM DTT, 50 mM NaCl, and 0.8 μL ATP at various concentrations were mixed and incubated at 28 °C for 20 min. The reaction was stopped by adding EDTA to a final concentration of 25 mM and 80 μL of dye solution. Then 10 μL of 30% sodium citrate was added and the absorbance at 620 nm was measured after 15 min by using a UV-VIS Spectrophotometer (TECAN, Shanghai, China). Lineweaver-Burk plots were drawn and the *K_m_* and *k_cat_* values were determined. To avoid exceeding the detection limit, the reactions to detect the NTPase activities of mutants and WT nsp10 were carried out as above except that the final concentrations of protein, dsDNA, and NTP were adjusted to 0.005 μM, 0.05 μM, and 0.4 mM, respectively.

### 2.7. Unwinding Assay

The reaction mixture (20 μL) containing 6 μM protein, 0.2 μM FAM labelled partially dsDNA described above, 2 μM unlabeled trap ssDNA (5′–GGTGGCGACGGCAGCGAGGC–3′), 40 mM HEPES pH 7.0, 50 mM NaCl, 2 mM MgCl_2_, 2 mM DTT, 0.01 mg/mL BSA, 0.02% Triton X-100 and 5% glycerol was incubated for 10 min at 28 °C. After the binding phase, unwinding was started by adding 2 mM NTP and incubating at 28 °C for 60 min. The reaction was stopped by addition of 5 μL loading buffer (100 mM Tris-HCl pH 7.5, 20 mM EDTA, 0.5% SDS, 0.1% bromophenol blue, and 0.1% Triton X-100). Samples were resolved by 10% native-PAGE running on ice. The gel was scanned with ChemiDoc MP Imaging System (BIO-RAD, Shanghai, China) at the wavelength of 520 nm.

### 2.8. Electrophoretic Mobility Shift Assay

EMSA assay was performed to detect the nucleic acid binding ability of PRRSV nsp10 and nsp10 mutants. The reaction mixture (20 μL) containing 4 μM protein, 2 μM FAM labelled partially dsDNA described above, 40 mM HEPES pH 7.0, 125 mM NaCl, 2 mM MgCl_2_, 2 mM DTT, 0.01 mg/mL BSA was incubated for 60 min at 28 °C. After incubation, 3 μL 50% glycerol was added to each sample to prepare for the electrophoretic mobility. The gel was scanned with ChemiDoc MP Imaging System (BIO-RAD) at the wavelength of 520 nm.

### 2.9. Model Generation

The model of PRRSV nsp10-ssDNA complex was generated based on the superposition of each individual domain of PRRSV nsp10 with that of EAV nsp10-DNA complex [23]. Minor manual adjustments were performed according to the calculations of SASREF [39].

### 2.10. Small-Angle X-ray Scattering

SAXS measurements of the nsp10 and nsp10-DNA complex were performed at the beamline BL19U2 of SSRF using previously published methods [40]. The complex was obtained by incubating 3.0 mg/mL nsp10 with ssDNA (5’–TTTTTTTTTTCGAGCACCGCTGCGGCTG–3’) in a 1:1.2 molar ratio at 4 °C overnight. The complex was further purified by SEC (Superdex 75 10/300 GL, GE Healthcare, Uppsala, Sweden). Software BioXTAS-RAW (Version 1.6.0) was used to process individual data [41]. Comparison of the scattering of nsp10 and complex model with SAXS experimental data was computed with FoXS [42].

## 3. Results

### 3.1. Overall Domain Organization of HP-PRRSV nsp10

**Figure 1:**
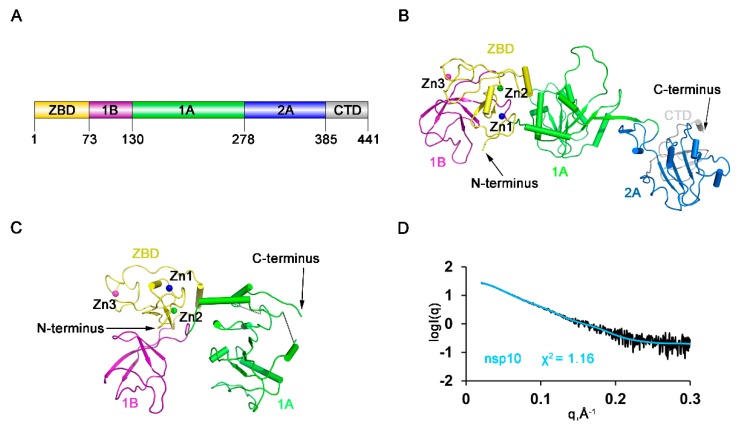
Overall structures of the full-length highly pathogenic porcine reproductive and respiratory syndrome virus (HP-PRRSV) nonstructural protein 10 (nsp10) and a truncated form. (**A**) Domain organization of PRRSV nsp10. PRRSV nsp10 contains an N-terminal zinc-binding domain (ZBD) (yellow), domain 1B (purple), two RecA-like domains 1A (green) and 2A (blue), and a C-terminal domain (CTD) (grey). Structures of (**B**) full-length nsp10 and (**C**) a truncated form lacking domains 2A and CTD. (**D**) Comparison of the calculated scattering profiles of nsp10 structure with small-angle X-ray scattering (SAXS) experimental data. Experimental data are represented in black.

The diffraction of the crystals of full-length HP-PRRSV nsp10 was poor after extensive optimization. However, after conducting the lysine methylation protocol [28], the resolution was greatly improved from 10 to 2.5 Å. Basic Local Alignment Search Tool (BLAST) searches of the PRRSV nsp10 sequence among the solved structures in the PDB database revealed that the sequence identities with known structures were low, with the highest value being only 30%. Moreover, the structure could not be solved using the closest homologs as search models after extensive trials by molecular replacement. We determined the structure by single-wavelength anomalous dispersion (SAD) phasing using the signals from the Zn atoms. Then we obtained crystals yielded from nsp10 incubated with a partially double-stranded DNA (dsDNA) or a single-stranded DNA (ssDNA) substrate. With the methylated nsp10 as a model, we determined the structures of full-length HP-PRRSV nsp10 and a truncated form (residues 4–273) by molecular replacement (Figure 1A–C; Table 1). However, no additional electron density for DNA could be identified. Comparison of these three structures showed that all the overall domain arrangement was similar (Figure S1A), except that the truncated form lacked the C-terminal 168 residues due to the degradation during the period of crystallization. Therefore, the full-length holo structure of HP-PRRSV nsp10 was used for further analysis. To differentiate HP-PRRSV nsp10 from LP-PRRSV nsp10, we will hereafter refer to the former as PRRSV nsp10 for simplicity, which was used throughout this study unless otherwise specified. The final model is composed of multiple functional domains (Figure 1A). The N-terminal ZBD has 13 conserved Cys/His residues, twelve of which participate in the coordination of three zinc ions. The middle helicase core belongs to the SF1 helicase family and consists of two RecA-like domains, referred to as 1A and 2A. Domain 1A folds as a parallel six-stranded β-sheet sandwiched between five and two α-helices on the sides, while domain 2A is comprised of a parallel four-stranded β-sheet and four α-helices. A characteristic β-barrel fold referred to as 1B consists of four β-strands arranged as two tightly packed anti-parallel β-sheets. We compared the crystal structure of PRRSV nsp10 with all structures in the PDB database using the Dali server [43]. The top two hits were different chains of EAV nsp10 complexed with DNA (PDB code 4N0O; Chain G/A; Z-score, 17.9/17.8; root mean square deviation (RMSD), 15.1/15.1 Å) and the third hit was EAV nsp10 (PDB code 4N0N; Z-score, 17.3; RMSD, 12.4 Å). We also compared each domain of PRRSV nsp10 with those of EAV nsp10 and MERS-CoV nsp13 (Table S1) and found that domains of PRRSV nsp10 were structurally more related to the domains of EAV nsp10 than to those of MERS-CoV nsp13. The ZBD domains 1B, 1A and 2A aligned well with their equivalents in EAV nsp10, with the Z-score of 6.9 (RMSD, 2.9 Å), 9.4 (RMSD, 1.8 Å), 17.5 (RMSD, 2.5 Å) and 18.0 (RMSD, 1.6 Å). However, the overall structure of EAV nsp10 is more compact, while PRRSV nsp10 adopts an extended structure. To rule out the possibility that the structure is a crystal stacking artefact, SAXS analysis was performed on PRRSV nsp10 (Figure 1D). The fit of the structure in solution with the crystal structure of PRRSV nsp10 gave a χ^2^ of 1.16 (Table S2). Hence the conformation of PRRSV nsp10 in solution is close to the one observed in the crystal structure. Taken together, these results reveal that PRRSV nsp10 adopts a different domain distribution relative to other helicases, such as EAV nsp10.

### 3.2. Biochemical Characterization of PRRSV nsp10

**Figure 2:**
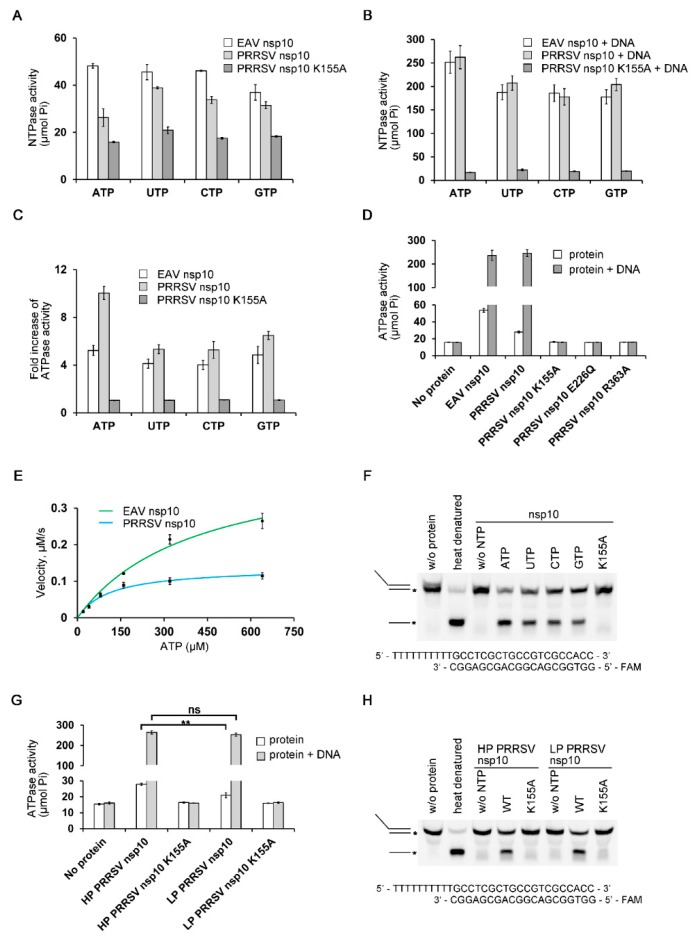
PRRSV nsp10 in vitro enzymatic activity assays. (**A**) NTPase activity of PRRSV nsp10 in the absence of nucleic acid substrate. HP-PRRSV nsp10 is called as PRRSV nsp10 unless otherwise specified. The final concentration of protein was 5 nM unless noted. (**B**) NTPase activity of PRRSV nsp10 in the presence of partially double-stranded DNA. (**C**) Effects of nucleic acid substrate on the ATPase activity of nsp10. (**D**) ATPase activities of different nsp10 variants compared with the activity of WT nsp10. (**E**) Determination of the kinetic parameters of ATPase reactions. A plot of velocity versus ATP concentration in ATPase reactions for nsp10 and equine arteritis virus (EAV) nsp10 is shown. The final concentrations of PRRSV nsp10 and EAV nsp10 were 20 nM. (**F**) Helicase assay shows that PRRSV nsp10 can utilize different NTPs to unwind dsDNA. (**G**) ATPase activity comparison of HP-PRRSV nsp10 and low pathogenic PRRSV (LP-PRRSV) nsp10. (**H**) Unwinding activity comparison of HP-PRRSV nsp10 and LP-PRRSV nsp10. No enzyme (*w*/*o* protein) and heat denatured controls are indicated. DNA strand with FAM label is marked with an asterisk. Error bars represent SD values from three separate experiments. ** *P* < 0.01; ns, not significant (Student’s unpaired *t*-test).

Since PRRSV nsp10 has an unusual extended structure comparing with EAV nsp10 (Figure S1B), we performed NTP hydrolysis and DNA unwinding assays to verify whether the recombinant protein was functionally active. PRRSV nsp10 could hydrolyze all NTPs and the activities could be stimulated by the presence of dsDNA (Figure 2A–C). We also performed a mutagenesis study focusing on key residues necessary for NTP binding and hydrolysis (Figure 2D). As expected, the mutations of three conserved residues, K155A within Walker A motif (motif I), E226Q within Walker B motif (motif II) and R363A within motif VI, abolished the ATPase activity [44,45]. This confirmed that the observed ATPase activity was completely attributed to PRRSV nsp10 used rather than to potential contaminations. Since the observed differences in different NTPs hydrolysis were minor, ATP was used in the experiments measuring the kinetic parameters of NTP hydrolysis. The data show that nsp10 displays ATPase activity with a turnover number (*k_cat_*) of 8.50 ± 1.17 s^–1^ and the catalytic efficiency (*k_cat_/K_m_*) of 0.047 ± 0.001 s^–1^·μM^–1^ (Figure 2E and Figure S2A). To determine whether the extended structure of PRRSV nsp10 influence ATPase, we also performed ATP hydrolysis assay with EAV nsp10 which exhibited ATPase activity with *k_cat_* = 27.60 ± 2.16 s^–1^ and *k_cat_/K_m_* = 0.045 ± 0.002 s^–1^·μM^–1^ (Figure 2E and Figure S2B). Although the catalytic efficiency of PRRSV nsp10 was comparable with that of EAV nsp10, PRRSV nsp10 was approximately threefold slower in hydrolyzing ATP. We speculated that PRRSV nsp10 had to change its non-productive conformation to hydrolyze NTP and the process resulted in the lower ATP hydrolysis velocity. Next, we assessed helicase activity with partial DNA duplex containing 5’ overhang. PRRSV nsp10 was able to utilize different NTPs to unwind the substrate (Figure 2F). Meanwhile, mutant K155A completely abolished helicase activity, indicating that the activity is dependent on NTP hydrolysis. In addition, we compared the enzymatic activities of HP-PRRSV and LP-PRRSV nsp10 (Figure 2G,H). The comparisons suggest that although the unwinding efficiency and ATPase activity of LP-PRRSV nsp10 were comparable with those of HP-PRRSV nsp10 in the presence of nucleic acid substrate, the latter was more efficient in hydrolyzing ATP without nucleic acid substrate. Taken together, our results demonstrate that PRRSV nsp10 expressed in bacteria is functionally active and shares the properties reported for helicases of other members of nidovirus.

### 3.3. Structure of the ZBD of PRRSV nsp10

**Figure 3:**
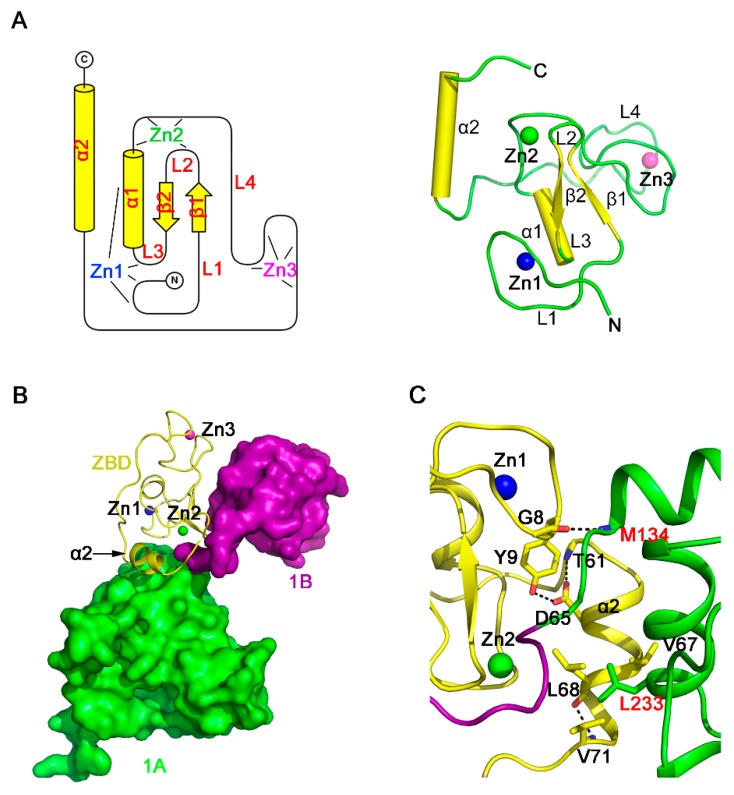
Structural characterization of the PRRSV nsp10 ZBD. (**A**) The topology (**left**) and ribbon model (**right**) of the ZBD. (**B**) Overview of the spatial orientation of the helix α2 of ZBD. Domains 2A and CTD are omitted for clarity. (**C**) Close-up view of the domain interface between ZBD and domain 1A. Residues engaged in interactions are shown as sticks. Domain colors are the same as in Figure 1A.

Our structure revealed that the ZBD of PRRSV nsp10 is a compact domain with three zinc-binding motifs stabilizing the fold (Figure 3A). The ZBD contains an N-terminal cross-braced module and a C-terminal treble-clef zinc finger (Figure S3A,B). The cross-braced module has two zinc ions. The first zinc ion (Zn1) is coordinated by three cysteine residues and one histidine residue (Cys7, Cys10, Cys25, and His28). Residues Cys7 and Cys10 are positioned at the loop L1, and Cys25 is located on the loop L3, while His28 comes from the α1 helix. The second zinc ion (Zn2) is coordinated by two cysteine residues and two histidine residues (Cys20, Cys35, His32, and His34). Residue Cys20 is cross-braced from the loop L2 in the first zinc finger, whereas Cys35, His32, and His34 are located on the loop L4 between helices α1 and α2. The N-terminal cross-braced module aligned with the RING-like module of EAV nsp10, with the Z-score of 5.1 (RMSD, 1.7 Å). The third zinc ion (Zn3) is coordinated by the C-terminal C3H type zinc finger, and the four chelated residues (Cys41, Cys50, Cys53, and His43) are from the loop L4. Superimposition of PRRSV nsp10 zinc-binding motifs and corresponding motifs in EAV nsp10 aligned with the Z-score of 5.4 and a RMSD of 2.2 Å (Figure S3C). Moreover, sequence alignment also demonstrates that the residues of the zinc-binding motifs are mostly conserved among PRRSV nsp10 and EAV nsp10 (Figure S3D). 

The ZBD of nidovirus helicases is important not only for the function but also for the interaction with other domains [23]. The EAV nsp10 ZBD affects the fold and activity of helicase core through extensive hydrophobic and hydrophilic interactions. The total interface area between ZBD and the helicase core is 769.9 Å^2^, as determined by the Protein Interfaces, Surfaces and Assemblies (PISA) server [46]. However, the corresponding area in PRRSV nsp10 is only 331.1 Å^2^. A major part of this interface involves the α2 helix (Figure 3B,C). The interface area between α2 and domain 1A is 188.1 Å^2^, smaller than the corresponding area in EAV nsp10 (535.4 Å^2^). Helix α2 interacts with the rest of the ZBD mainly through hydrophilic interactions, while interacting with domain 1A through hydrophobic interactions. Besides, the ZBD interacts with domain 1A through the hydrogen bond formed by main chains of Gly8 and Met134. ZBD may also interact with other viral proteins or cellular proteins [23,24,47], since having a putative protein interaction surface similar to the protein-binding surface of the Upf1 CH domain [48], which is also found in EAV and MERS-CoV. However, this hydrophobic pocket on the surface of PRRSV nsp10 ZBD is covered by the domain 1B, which may affect the function of ZBD (Figure S4).

### 3.4. The Novel CTD Essential for the Overall Structure and Enzymatic Activities

**Figure 4:**
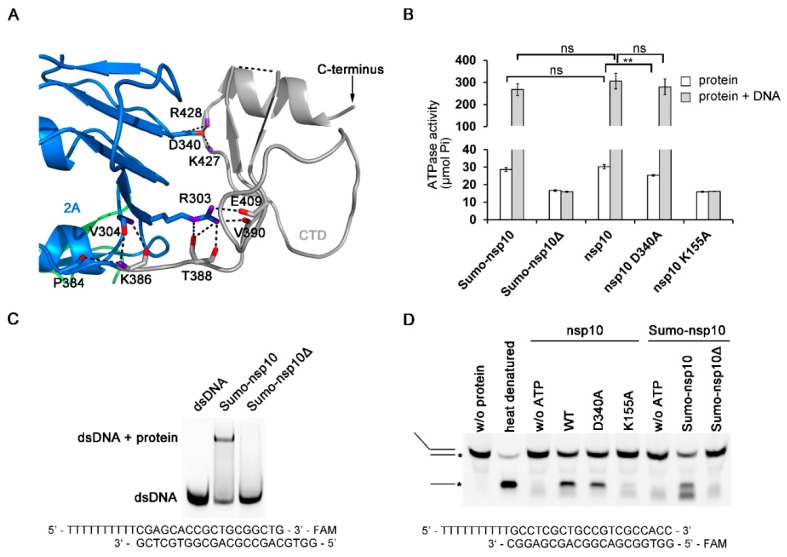
Characterization of the PRRSV nsp10 CTD. (**A**) Close-up view of the domain interface between CTD and domain 2A. Residues engaged in interactions are shown as sticks. Domain colors are the same as in Figure 1A. (**B**) ATPase activities of PRRSV variants. The final concentrations of PRRSV nsp10 variants were 5 nM. (**C**) The binding affinity of PRRSV nsp10Δ to dsDNA is demonstrated through EMSA. (**D**) Helicase assay shows that PRRSV nsp10Δ cannot unwind dsDNA. No enzyme (w/*o* protein) and heat denatured controls are indicated. DNA strand with FAM label is marked with an asterisk. Error bars represent SD values from three separate experiments. ** *P* < 0.01; ns, not significant (Student’s unpaired *t*-test).

The CTD adopts a novel fold as a search of the PDB using the Dali server revealed no significant similarity to other domains (Figure 4A). The sequence conservation analysis showed that the CTD is poorly conserved among the family *Arteriviridae* and absent in family *Coronaviridae* [23,24]. Since the PRRSV nsp10 structure shows that the CTD is a flexible domain, we then tried to design truncated constructs to find out the role of the CTD in regulating enzymatic activities. We cloned and expressed nine truncated variants that are based on the domain boundary (Table S3). Only one truncated form (residues 1–366) which contained all characteristic SF1 helicase motifs was soluble upon cell lysis. For simplicity, we hereafter referred to this truncated protein as PRRSV nsp10Δ, which was used throughout this study. nsp10Δ could not be expressed in soluble form, lacking the CTD resulted in protein degradation, unless by fusing this truncated protein to SUMO tag. We performed ATP hydrolysis assay, EMSA, and DNA unwinding assay to verify whether nsp10Δ was enzymatically active. The results revealed that nsp10Δ completely abolished ATPase, DNA binding activity, and consequentially also dsDNA unwinding activity (Figure 4B–D). To rule out the possibility that the potential steric clashes between the SUMO tag and PRRSV nsp10 resulted in the deficiencies in the enzymatic activities, we also evaluated the enzymatic activities of Sumo tagged full-length PRRSV nsp10 (Sumo-nsp10). It was found that the enzymatic activity of Sumo-nsp10 was comparable with that of the protein with an authentic N terminus (Figure 4B,D and Figure S5A). Thus, the differences in function between nsp10 and nsp10Δ were more due to the lack of CTD. To find out the role of CTD in regulating the enzymatic activities, we performed a mutagenesis study. The interface area between domain 2A and CTD is 622.2 Å^2^, mainly contributed by hydrophilic interactions (Figure 4A). For instance, both Lys427 and Arg428 form hydrogen bonds with Asp340, which is a conserved residue of motif Va coupling between NTP and nucleic acid binding sites [49,50]. The mutant variant D340A displayed reduced ATPase activity (Figure 4B). However, the mutation had no obvious influence on the ATPase activity in the presence of nucleic acid substrate, suggesting that the reduction in ATPase activity caused by the mutation in the interface area between domain 2A and CTD could be offset by the stimulation of nucleic acids. Thus, we speculated that the deficiencies in the enzymatic activities of nsp10Δ might be due to a misfolded protein, rather than due to lack of a critical domain. Taken together, these results suggest that CTD may stabilize the fold of nsp10 and regulate enzymatic activities.

### 3.5. Regions Critical for Enzymatic Activities

**Figure 5:**
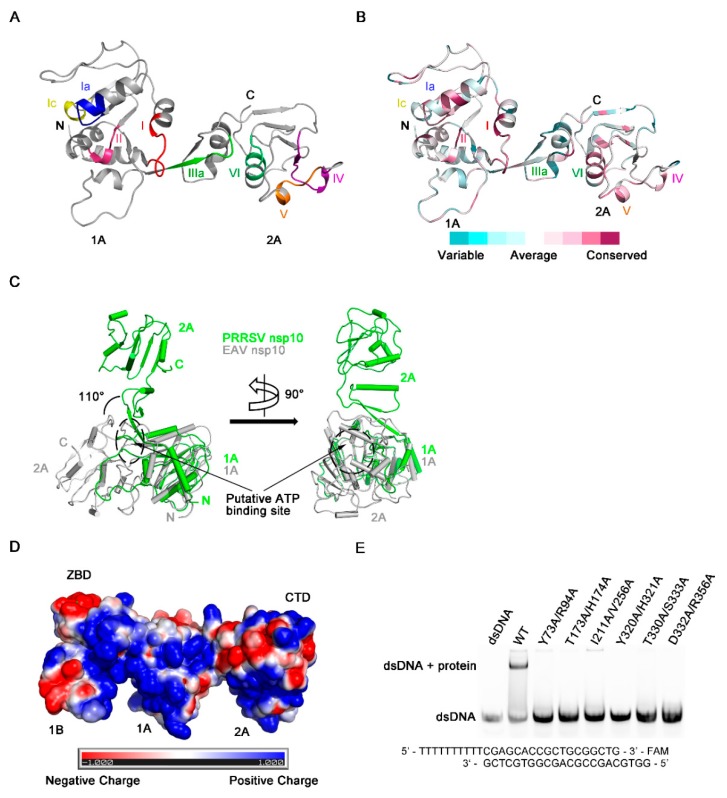
Regions critical for enzymatic activities. (**A**) Position of conserved motifs necessary for NTP binding, hydrolysis and nucleotide binding in the structure of the PRRSV nsp10 helicase core. (**B**) The helicase core was colored by conservation calculated using Consurf [52] based on multiple sequence alignment of PRRSV nsp10, EAV nsp10, MERS-CoV nsp13, severe acute respiratory syndrome coronavirus nsp13, and lactate dehydrogenase-elevating virus nsp10 [23,24,25,47,53,54]. (**C**) Structural comparison of the helicase core of PRRSV nsp10 and EAV nsp10 reveals differences in structural rearrangements. These two structures are superimposed on 1A domains. The putative ATP binding site is shown as a dotted circle. A 90° rotation view is shown in the right panel. (**D**) Overview of surface charges of PRRSV nsp10. (**E**) The binding abilities of PRRSV nsp10 mutants to dsDNA are demonstrated through EMSA.

**Figure 6:**
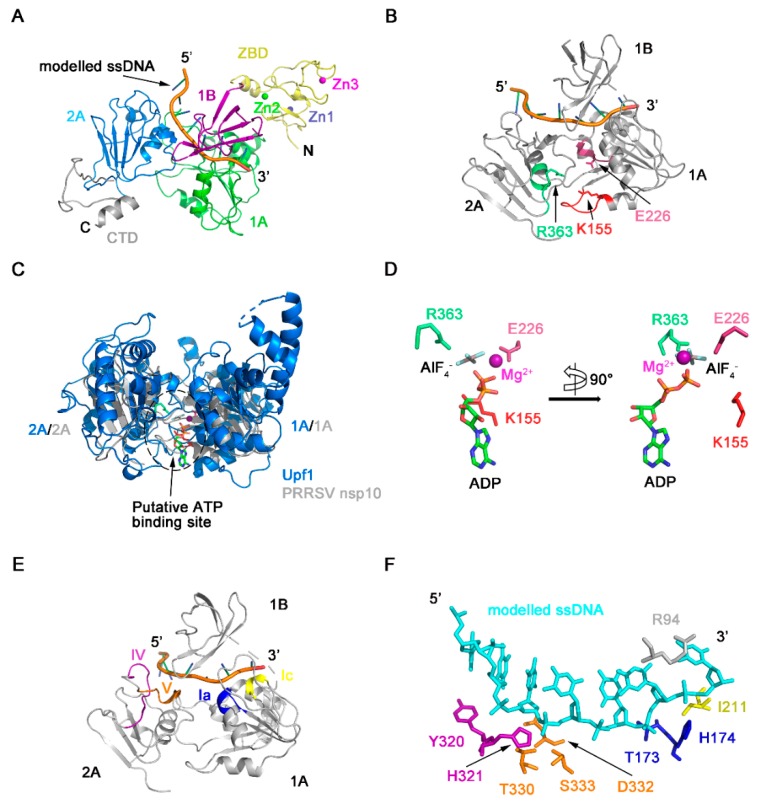
Model of PRRSV nsp10-DNA complex. (**A**) Model of PRRSV nsp10-DNA complex was generated based on the structure of EAV nsp10-DNA complex. The rest part of ssDNA is omitted for clarity. Domain colors are the same as in Figure 1A. (**B**) Position of conserved motifs and key residues necessary for NTP binding and hydrolysis in the structure of the nsp10-DNA complex model. (**C**) Superposition between helicase cores of human Upf1-ADP-AlF_4_^-^ (PDB ID: 2XZO) in marine and PRRSV nsp10-DNA complex model in grey. These two structures are superimposed on 2A domains. (**D**) Close-up view of the modelled ADP-AlF_4_^-^ with the residues of PRRSV nsp10, which involved in NTP binding and hydrolysis. A 90° rotation view is shown in the right panel. Motifs colors are the same as in Figure 5A. (**E**) Position of conserved motifs necessary for nucleotide binding in the structure of the nsp10-DNA complex model. The ZBD, the CTD, and the rest part of ssDNA in the complex model are omitted for clarity. (**F**) Close-up view of the modelled nucleic acids with the residues involved in nucleic acids’ binding. Motifs colors are the same as in Figure 5A.

NTP hydrolysis assays demonstrated PRRSV nsp10 could hydrolyze different NTPs (Figure 2A). The NTP binding pocket of SF1 helicases located between domains 1A and 2A includes several conserved motifs—motifs I, II, IIIa, and VI, which are well conserved in PRRSV nsp10 (Figure 5A,B). These four conserved motifs should form a cavity to accommodate NTP. However, in our solved PRRSV nsp10 structure, although mutants of key residues failed to hydrolyze ATP, these four motifs line up since domain 2A rotates about 110 degrees relative to domain 1A comparing with domain 2A of EAV nsp10 (Figure 5C). Besides, due to this large rotation of domain 2A, motifs devoted to nucleic acid binding are far apart instead of being located on the opposite face of the helicase core relative to the nucleotide binding site, including motifs Ia, Ic, IV, and V (Figure 5A). This conformational arrangement inhibits the formation of a nucleic acid-binding channel involving both helicase core domains and domain 1B. Surface electrostatic potential also demonstrates that domains 1A and 2A in the solved PRRSV nsp10 structure cannot form a positively charged channel to accommodate nucleic acid (Figure 5D). Since PRRSV nsp10 does bind nucleic acid substrates in vitro (Figure S5A,B), we speculate that PRRSV nsp10 would undergo a conformational change when forming contacts with substrates. Thus we generated a model of PRRSV nsp10-ssDNA complex based on the superposition of each individual domain of PRRSV nsp10 with that of the EAV nsp10-DNA complex (Figure 6A and Figure S6). The model shows that motifs I, II, and VI together with the residues essential for NTP recognition and hydrolysis surround the cleft between domains 1A and 2A (Figure 6B). To verify the complex model, we superimposed it onto the structure of the helicase core of human Upf1-ADP-AlF_4_^−^ [51]. The ATP analogue is accommodated well by the cleft (Figure 6C). The key residues necessary for NTPase activity were located close to the modeled ATP analogue and were conserved between PRRSV nsp10 and Upf1 (Figure 6D). Meanwhile, motifs Ia, Ic, IV, and V together with the domain 1B form a nucleic acid-binding channel in which the 5’ and 3’ ends of the substrate are located in domains 2A and 1A, respectively (Figure 6E). The residues essential for DNA-recognizing in EAV nsp10, e.g., the histidine in motif Ia, the tyrosine in motif IV, and the threonine in motif V, are well conserved in PRRSV nsp10 and located close to the modeled ssDNA (Figure 6F). To verify our complex model, we constructed six double mutants including Y73A/R94A, T173A/H174A, I211A/V256A, Y320A/H321A, T330A/S333A, and D332A/R356A at the binding interface. We detected their binding activity for dsDNA using EMSA and all the mutants failed to bind the substrate (Figure 5E). Furthermore, we performed SAXS experiments. PRRSV nsp10-DNA complex was obtained by incubating PRRSV nsp10 with ssDNA and demonstrated by SEC (Figure S5C). The fit of the scattering of the complex model with experimental data gave a χ^2^ of 2.6 (Figure S7; Table S2), significantly better than the fit of holo nsp10. Meanwhile, the latter had an extreme value of c2 parameter which indicated data overfitting. Taken together, the similarities in the motif sequences and locations between the PRRSV nsp10-DNA model and EAV nsp10-DNA complex structure suggest that PRRSV nsp10 might recognize substrates via a similar mechanism.

## 4. Discussion

Our crystallization trials with the unliganded PRRSV nsp10 yielded poor-quality crystals. Owing to the incubation with DNA which greatly improved the diffraction, we have successfully solved the structure of holo HP-PRRSV nsp10. Structural comparisons of the individual domains of PRRSV nsp10 with those of EAV nsp10 and MERS-CoV nsp13 show that the domain organization is conserved throughout nidovirus helicases. Meanwhile, PRRSV nsp10 is structurally more related to EAV nsp10 than to MERS-CoV nsp13. However, our study reveals a number of significant differences compared with its closest homolog, EAV nsp10. Firstly, PRRSV nsp10 has a much smaller interface area between ZBD and the helicase core, as well as the putative protein interaction surface which is covered by domain 1B. Since ZBD can regulate enzymatic activities, it is not surprising that PRRSV nsp10 hydrolyzed ATP more slowly. Secondly, the C-terminal treble-clef zinc finger of EAV nsp10 is stabilized by multiple hydrogen-bonding interactions, while the corresponding zinc finger of PRRSV nsp10 is more flexible without such an extensive array of hydrogen bonds, suggesting that the C-terminal zinc-binding motif may have less effect on the enzymatic activities in PRRSV nsp10 than in EAV nsp10. This speculation is confirmed by previous mutagenesis studies that H44A of EAV nsp10 (ligand for Zn3) retained a limited level of ATPase and helicase activities while the mutations in the mutant variant H43A had no obvious influence on the enzymatic activities of PRRSV nsp10 [55,56]. Thirdly, the resolution of the crystals of full-length EAV nsp10 was poor after extensive optimization and diffracting crystals could only be obtained for a truncated form lacking the CTD. However, the truncated EAV nsp10 had a lower unwinding activity and a higher ATPase activity. In contrast, the folding of PRRSV nsp10 was disrupted without the CTD. Although fusing to the SUMO tag could stabilize the fold of nsp10Δ, this recombinant protein lacks enzymatic activities. Structural analysis revealed that the interaction between domain 2A and the CTD was strong, which also suggested that the CTD is essential to stabilize the fold of nsp10 and regulate enzymatic activities. Taken together, these results suggest that CTD may play different roles in regulating enzymatic activities in different nidovirus helicases, since this domain is poorly conserved. Last but not least, the most striking difference is the domain arrangement. The overall structure of EAV nsp10 is more compact, and the domains 1A and 2A form the cleft to accommodate NTP. Meanwhile, these two domains together with the domain 1B form the channel to bind nucleic acid. However, we could not find the cleft or the channel in the PRRSV nsp10 because the involved motifs are far apart although they are conserved. This non-productive conformation could change profoundly induced by DNA binding. Thus, we generated a complex model according to the SAXS data and the EAV nsp10-DNA complex. The similarities in the sequences and locations of key residues critical for enzymatic activities between the PRRSV nsp10-DNA model and EAV nsp10-DNA complex structure suggest that PRRSV nsp10 might recognize substrates via a similar mechanism. Thus, we put forward several hypotheses to account for such an apparent non-productive conformation of holo PRRSV nsp10. Firstly, according to the previous research, simultaneous synthesis and unwinding of the template strand would lead to collision due to opposite polarities of nsp9 and nsp10. Thus, an enzymatically silent form of nsp10 may be necessary for the discontinuous negative-strand RNA synthesis according to the hypotheses which explained the cooperativity between nsp9 and nsp10 [50,57]. Secondly, PRRSV nsp10 may abolish unwinding activity since its potential role in post-transcriptional quality control. It was speculated that nidovirus helicases could be involved in process targeting aberrant viral transcripts to prevent the synthesis of potentially harmful proteins [23]. Besides, PRRSV nsp10 may reduce ORF1ab transcription and is hypothesized to negatively regulate the expression of other host and viral proteins [58]. Hence, it is not surprising that PRRSV nsp10 adopts such an unusual arrangement to avoid dysfunction. Thus, further experiments are needed to test the above hypotheses. Meanwhile, strains of PRRSV-2 cause diseases from mild ones to fatal ones. The helicase region can vary up to 8% on the amino acid level between type 2 strains [21]. Thus we did functional analyses of HP versus LP-PRRSV strains. HP-PRRSV nsp10 was more efficient in hydrolyzing ATP in the absence of nucleic acid substrate. Therefore, further investigations are needed to study the relationship between strains variations and enzymatic activities.

In summary, our analyses demonstrate that while the structures of the individual domains of PRRSV nsp10 are closely related to their equivalents in EAV nsp10 and MERS-CoV nsp13, PRRSV nsp10 adopts an extended domain arrangement confirmed by crystal structures and SAXS. However, DNA binding could induce a profound conformational change of nsp10, resulting in a conformation more similar to the structure of the EAV nsp10-DNA complex. Nevertheless, the exact mechanisms of the conformational change remain undefined, which requires further experimental investigation. Finally, our results provide the first structural insight into the helicase of PRRSV essential for structure-based drug design.

## Figures and Tables

**Table 1:** Data collection and refinement statistics of PRRSV nsp10.

Parameters		Methylated PRRSV nsp10 (6JDR)	PRRSV nsp10 (6JDU)	Truncated PRRSV nsp10 (6JDS)
Data collection	Wavelength	0.979	0.979	0.979
	Space group	*P4_3_2_1_2*	*P4_3_2_1_2*	*P4_3_2_1_2*
Cell dimensions	a, b, c (Å)	93.16, 93.16, 148.11	92.91, 92.91, 147.70	99.98, 99.98, 83.37
	α, β, γ (°)	90, 90, 90	90, 90, 90	90, 90, 90
	Resolution (Å) ^1^	50.00–2.50 (2.54–2.50)	50.00–2.80 (2.85–2.80)	50.00–2.50 (2.54–2.50)
	*R*merge (%)	9.9 (85.4)	11.9 (70.3)	8.0 (82.7)
	*I*/σ	31.1 (2.5)	19.9 (2.7)	36.4 (4.8)
	Completeness (%)	99.9 (99.9)	97.7 (81.9)	99.6 (93.6)
	Total No. of reflections	1492609	1424962	518654
	Unique reflections	23333	16581	15236
	Redundancy	64.0	85.9	34.0
Refinement	Resolution (Å)	50.00–2.50 (2.56–2.50)	50.00–2.81 (2.88–2.81)	50.00–2.50 (2.57–2.50)
	No. of reflections	21997 (1513)	15074 (877)	14161 (853)
	*R*_work_/*R*_free_ (%)	23.5/27.2	22.5/23.8	21.1/23.1
	No. of atoms			
	Protein	3254	3162	1908
	Ligand/ions	10	6	4
	Water	193	168	174
*B*-factors (Å^2^)	Protein	65.9	51.0	38.7
	Ligand/ion	65.5	39.0	30.9
	Water	55.0	35.0	38.1
rms deviations	Bond lengths (Å)	0.004	0.007	0.004
	Bond angles (°)	0.897	1.147	0.846
	Ramachandran Plot (%) ^2^	96.7/2.8/0.5	96.2/3.3/0.5	97.6/2.4/0

^1^ Statistics for highest resolution shell. ^2^ Residues in favored regions, allowed regions, outliers in Ramachandran plot.

## Supplementary Materials

The following are available online at https://www.mdpi.com/1999-4915/12/2/215/s1, Figure S1: Structural comparisons of methylated nsp10, truncated nsp10, PRRSV nsp10, and EAV nsp10. Figure S2: ATPase activities of PRRSV nsp10 and EAV nsp10 were determined from Lineweaver-Burk plots of hydrolysis activity using the malachite green assay. Figure S3: Structural characterization of the PRRSV nsp10 ZBD. Figure S4: Putative protein interaction surface of the nsp10 ZBD. Figure S5: Validation of the nucleic acid binding ability of PRRSV nsp10. Figure S6: Structural comparison of PRRSV nsp10-DNA complex and holo PRRSV nsp10. Figure S7: Comparison of holo nsp10 and complex model with SAXS experimental data of nsp10-DNA complex. Table S1: Pairwise comparison of the isolated ZBD/CH, 1B, 1A/RecA1, 2A/RecA2 and helicase core (1A/RecA1&2A/RecA2) domains of PRRSV nsp10, EAV nsp10 and MERS-CoV nsp13. Table S2: SAXS data collection and analysis. Table S3: Examples of insoluble constructs.
