# Supplementary material for: Helicase of Type 2 Porcine Reproductive and Respiratory Syndrome Virus Strain HV Reveals a Unique Structure
Source: Viruses. 2020 Feb 14;12(2):215. doi: 10.3390/v12020215 (PMC7077220; doi:10.3390/v12020215)
Supplement: Supplementary file 1 [file viruses-12-00215-s001.pdf]

# Supplementary Materials

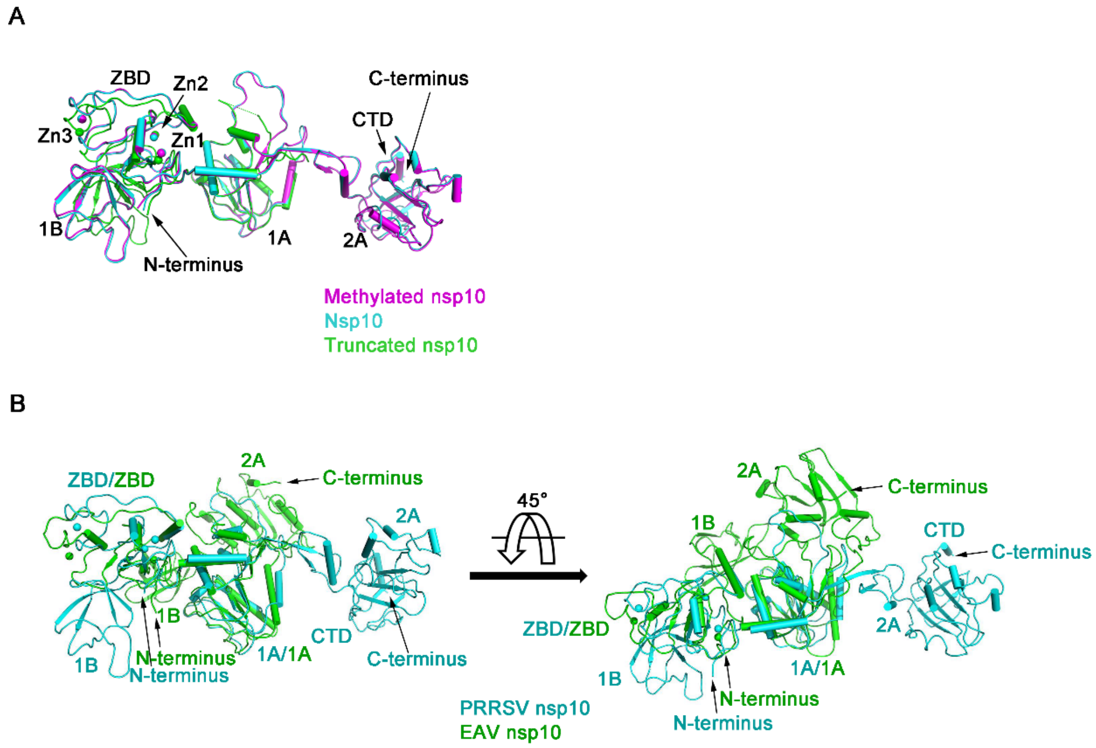


**Figure S1** Structural comparisons of (**A**) methylated nsp10 (magenta), nsp10 (cyan), and truncated nsp10 (green) and (**B**) PRRSV nsp10 (cyan) and EAV nsp10 (green). A 45° rotation view is shown in the right panel. The structures are superimposed on 1A domains.

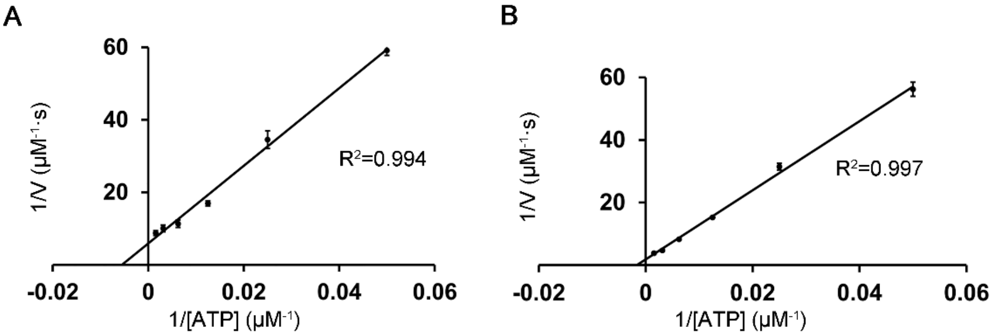


**Figure S2** ATPase activities of (**A**) PRRSV nsp10 and (**B**) EAV nsp10 were determined from Lineweaver-Burk plots of hydrolysis activity using the malachite green assay. Error bars represent SD values from three separate experiments.

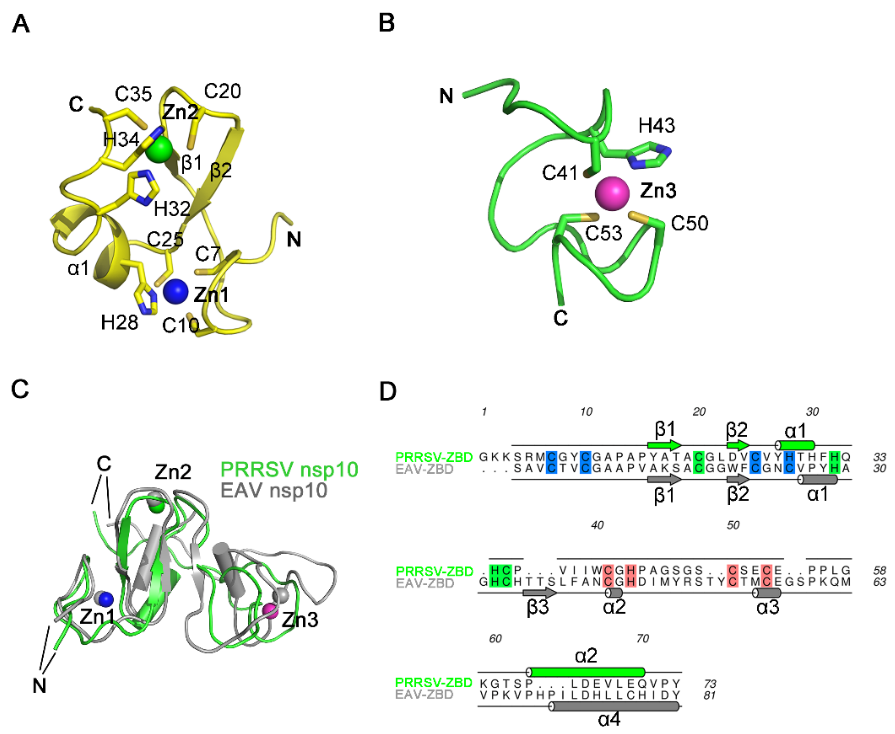


**Figure S3** Structural characterization of the PRRSV nsp10 ZBD. (**A**) Structure of the cross-braced zinc finger and (**B**) treble-clef zinc finger. The residues coordinating the $Zn^{2+}$ ions are shown as sticks. (**C**) Superposition of the ZBD of PRRSV nsp10 (green) and EAV nsp10 (PDB code: 4N0N; grey). (**D**) Sequence alignment of ZBD of PRRSV nsp10 and EAV nsp10. Multiple sequence alignment was carried out using Clustal Omega [1]. The illustration of sequence alignment was generated using ALINE [2]. Minor manual adjustments were performed in accordance with the superimposition.

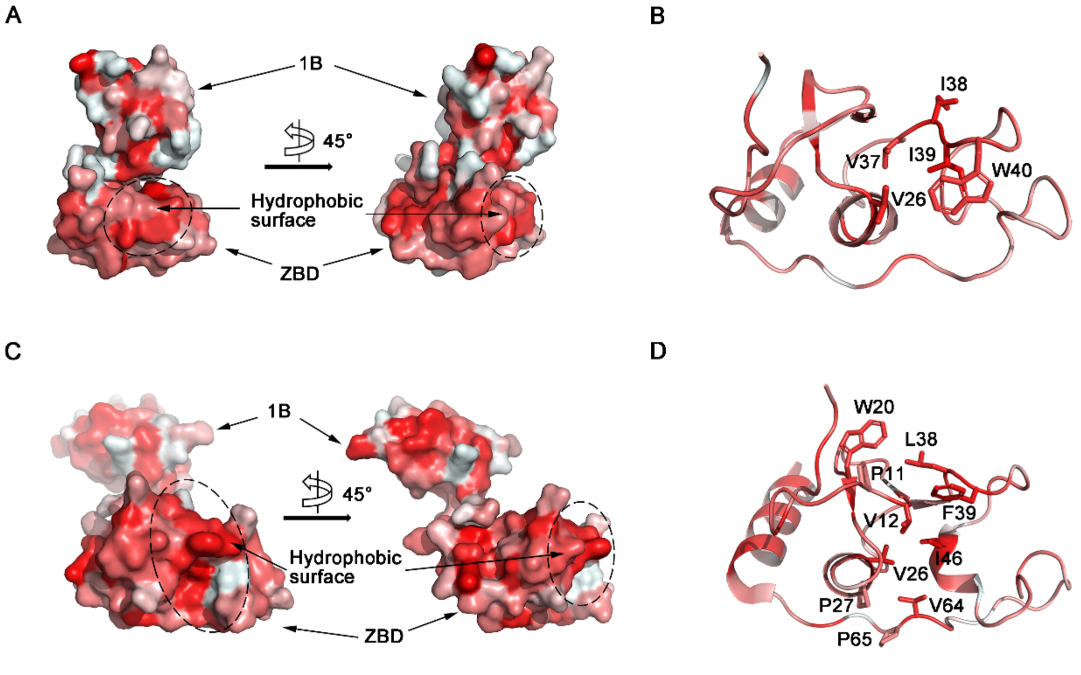


**Figure S4** Putative protein interaction surface of the nsp10 ZBD. Hydrophobic surface of (**A**) the PRRSV nsp10 ZBD and (**C**) the EAV nsp10 ZBD. The hydrophobic pocket is

covered by domain 1B in PRRSV nsp10. A 45° rotation view is shown in the right panel. Close-up view of hydrophobic pockets of (**B**) PRRSV nsp10 and (**D**) EAV nsp10. Domain 1B is omitted for clarity. Hydrophobic residues are shown as sticks. The orientation is the same as in panels A and C. Colors are calculated according to the method (https://pymolwiki.org/index.php/ Color_h).

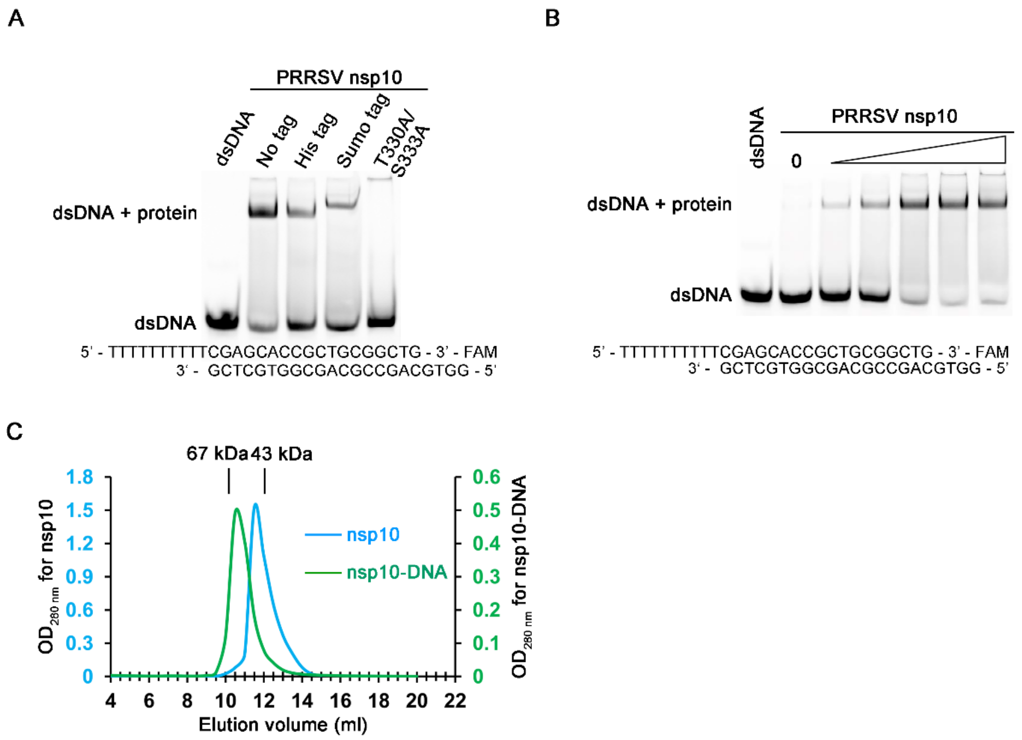


**Figure S5** Validation of the nucleic acid binding ability of PRRSV nsp10. (**A**) The binding abilities of PRRSV nsp10 or mutant with different affinity tags to dsDNA are demonstrated through EMSA. (**B**) The binding ability of nsp10 with an authentic N terminus to dsDNA is demonstrated through EMSA. (**C**) SEC profile of nsp10 in the absence (cyan) or presence (green) of ssDNA (Superdex 75 10/300 GL column, GE Healthcare, Uppsala, Sweden). Elution volumes of the molecular mass standards are marked at the top of the panel. dsDNA, partially double-stranded DNA (the sequences are shown in the figures); WT, no tagged protein; T330A/S333A, no tagged T330A/S333A mutated protein; His-nsp10, His-tagged nsp10; Sumo, Sumo-tagged nsp10.

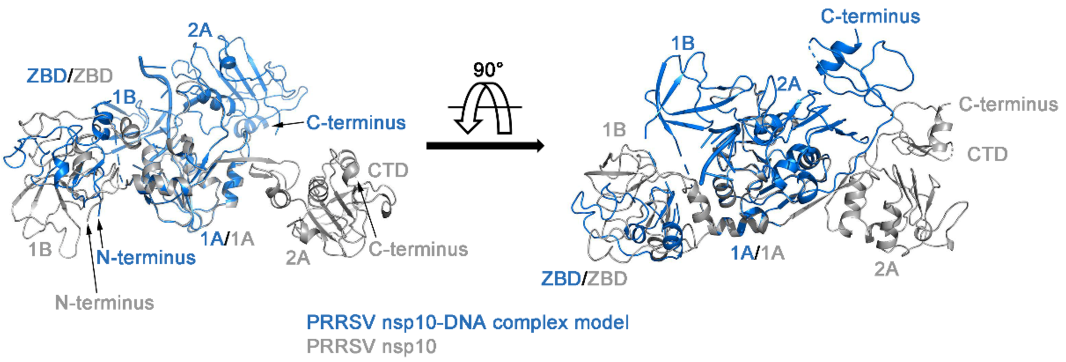


**Figure S6** Structural comparison of PRRSV nsp10-DNA complex model (marine) and holo PRRSV nsp10 (grey). The structures are superimposed on 1A domains. A 90° rotation view is shown in the right panel.

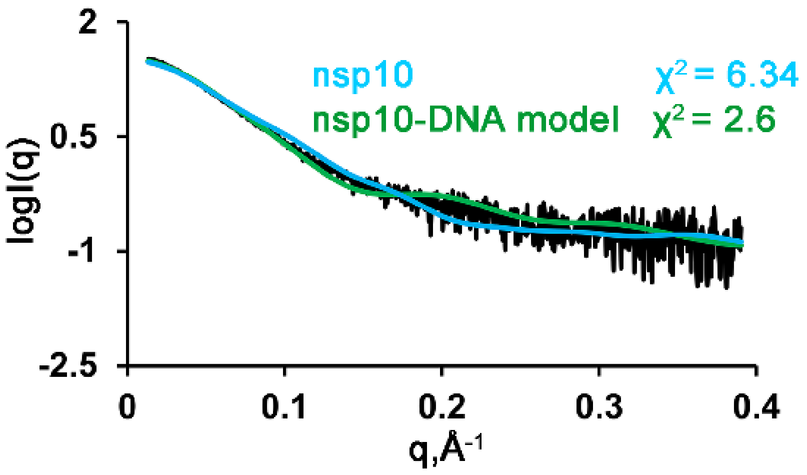


**Figure S7** Comparison of holo nsp10 and complex model with SAXS experimental data of nsp10-DNA complex. The model of PRRSV nsp10-DNA complex was generated based on the superimposition of each individual domain of PRRSV nsp10 with that of EAV nsp10. Experimental data are represented in black.

**Table S1.** Pairwise comparison of the isolated ZBD/CH, 1B, 1A/RecA1, 2A/RecA2 and helicase core (1A/RecA1&2A/RecA2) domains of PRRSV nsp10, EAV nsp10, and MERS-CoV nsp13[1].

| Domain | Comparison | Dali Z-score | RMSD (Å) |
|---|---|---|---|
| **ZBD/CH** | PRRSV vs EAV | 6.9 | 2.9 |
| | PRRSV vs MERS-CoV | 3.3 | 3.0 |
| | EAV vs MERS-CoV | 4.2 | 3.4 |
| **1B** | PRRSV vs EAV | 9.4 | 1.8 |
| | PRRSV vs MERS-CoV | 2.6 | 2.7 |
| | EAV vs MERS-CoV | 3.2 | 2.4 |
| **1A/RecA1** | PRRSV vs EAV | 17.5 | 2.5 |
| | PRRSV vs MERS-CoV | 10.4 | 3.0 |
| | EAV vs MERS-CoV | 14.1 | 2.5 |
| **2A/RecA2** | PRRSV vs EAV | 18.0 | 1.6 |
| | PRRSV vs MERS-CoV | 10.6 | 2.4 |
| | EAV vs MERS-CoV | 10.6 | 2.3 |
| **Helicase Core** | PRRSV vs EAV | 19.0 | 12.9 |
| **(1A/RecA1&2A/RecA2)** | PRRSV vs MERS-CoV | 11.4 | 11.4 |
| | EAV vs MERS-CoV | 22.1 | 2.5 |

[1] PRRSV nsp10, PDB ID: 6JDU. EAV nsp10, PDB ID: 4N0N. MERS-CoV nsp13, PDB ID: 5WWP.

**Table S2.** SAXS data collection and analysis.

| Parameters | PRRSV nsp10 | PRRSV nsp10-DNA |
|---|---|---|
| **Data collection** | | |
| Instrument | SSRF BL19U2 | SSRF BL19U2 |
| Wavelength (Å) | 1.003 | 1.030 |
| Exposure time (s) | 1.0 | 1.0 |
| Concentration (mg/ml) | 3.0 | 0.5 |
| Temperature (K) | 283 | 283 |
| **Structural parameters** | | |
| $I(0)$ (from $P(r)$) | 31.8 | 35.9 |
| $R_g$ (Å) (from $P(r)$) | 32.7 | 36.6 |
| $I(0)$ (from Guinier) | 30.8 | 35.7 |
| $R_g$ (Å) (from Guinier) | 30.5 | 35.5 |
| $D_{max}$ (Å) | 104 | 119 |
| Porod volume $V_p$ (Å$^3$) | 87306.6 | 99132.0 |
| Correlation volume $V_c$ (Å$^2$) | 418.2 | 507.0 |
| **Molecular mass** | | |
| Mass (kDa)[1] | 49.0 | 57.5 |
| Mass (kDa)[2] | 44.6 | 58.8 |
| Mass (kDa)[3] | 46.5 | 58.8 |
| **Data processing** | | |
| Data reduction and processing | BioXTAS-RAW | BioXTAS-RAW |
| Validation | FoXS | FoXS |
| $\chi^2$ | 1.16 | 2.60 |

| | | |
|---|---|---|
| c1, c2 | 1.05, 0.83 | 1.05, 2.33 |

[1] Mass was calculated from protein sequence

[2] Mass was calculated from Porod volume $V_p$

[3] Mass was calculated from correlation volume $V_c$

**Table S3.** Examples of insoluble constructs.

| No. | Construct boundaries | Affinity/Solubility tag |
|---|---|---|
| 1 | G1 – R365 | N-terminal $His_6$ |
| 2 | G1 – D372 | N-terminal $His_6$ |
| 3 | G1 – H374 | N-terminal $His_6$ |
| 4 | G1 – R375 | N-terminal $His_6$ |
| 5 | G1 – D382 | N-terminal $His_6$ |
| 6 | G1 – A385 | N-terminal $His_6$ |
| 7 | G1 – K386 | N-terminal $His_6$ |
| 8 | G1 – H395 | N-terminal $His_6$ |

# Supplementary References

1 Madeira, F.; Park, Y.M.; Lee, J.; Buso, N.; Gur, T.; Madhusoodanan, N.; Basutkar, P.; Tivey, A.R.N.; Potter, S.C.; Finn, R.D.; et al. The EMBL-EBI search and sequence analysis tools APIs in 2019. *Nucleic Acids Res.* **2019**, *47*, W636–W641, doi:10.1093/nar/gkz268.

2 Bond, C.S.; Schuttelkopf, A.W. ALINE: a WYSIWYG protein-sequence alignment editor for publication-quality alignments. *Acta Crystallogr. D Biol. Crystallogr.* **2009**, *65*, 510–512, doi: 10.1107/S0907444909007835.
